# Validation of potential RNA biomarkers for prostate cancer diagnosis and monitoring in plasma and urinary extracellular vesicles

**DOI:** 10.3389/fmolb.2023.1279854

**Published:** 2023-11-30

**Authors:** Agnese Brokāne, Cristina Bajo-Santos, Pawel Zayakin, Alberts Belovs, Juris Jansons, Vilnis Lietuvietis, Elena S. Martens-Uzunova, Guido W. Jenster, Aija Linē

**Affiliations:** ^1^ Latvian Biomedical Research and Study Centre, Riga, Latvia; ^2^ Riga Stradiņš University, Riga, Latvia; ^3^ Department of Urology, University Medical Center Rotterdam, Rotterdam, Netherlands

**Keywords:** prostate cancer, extracellular vesicles, RNA biomarkers, liquid biopsies, droplet digital PCR

## Abstract

**Introduction:** Prostate cancer (PCa), one of the most prevalent malignancies affecting men worldwide, presents significant challenges in terms of early detection, risk stratification, and active surveillance. In recent years, liquid biopsies have emerged as a promising non-invasive approach to complement or even replace traditional tissue biopsies. Extracellular vesicles (EVs), nanosized membranous structures released by various cells into body fluids, have gained substantial attention as a source of cancer biomarkers due to their ability to encapsulate and transport a wide range of biological molecules, including RNA. In this study, we aimed to validate 15 potential RNA biomarkers, identified in a previous EV RNA sequencing study, using droplet digital PCR.

**Methods:** The candidate biomarkers were tested in plasma and urinary EVs collected before and after radical prostatectomy from 30 PCa patients and their diagnostic potential was evaluated in a test cohort consisting of 20 benign prostate hyperplasia (BPH) and 20 PCa patients’ plasma and urinary EVs. Next, the results were validated in an independent cohort of plasma EVs from 31 PCa and 31 BPH patients.

**Results:** We found that the levels of NKX3-1 (*p* = 0.0008) in plasma EVs, and tRF-Phe-GAA-3b (*p* < 0.0001) tRF-Lys-CTT-5c (*p* < 0.0327), piR-28004 (*p* = 0.0081) and miR-375-3p (*p* < 0.0001) in urinary EVs significantly decreased after radical prostatectomy suggesting that the main tissue source of these RNAs is prostate and/or PCa. Two mRNA biomarkers—GLO1 and NKX3-1 showed promising diagnostic potential in distinguishing between PCa and BPH with AUC of 0.68 and 0.82, respectively, in the test cohort and AUC of 0.73 and 0.65, respectively, in the validation cohort, when tested in plasma EVs. Combining these markers in a biomarker model yielded AUC of 0.85 and 0.71 in the test and validation cohorts, respectively. Although the PSA levels in the blood could not distinguish PCa from BPH in our cohort, adding PSA to the mRNA biomarker model increased AUC from 0.71 to 0.76.

**Conclusion:** This study identified two novel EV-enclosed RNA biomarkers–NKX3-1 and GLO1–for the detection of PCa, and highlights the complementary nature of GLO1, NKX3-1 and PSA as combined biomarkers in liquid biopsies of PCa.

## Introduction

PCa is the second leading cause of cancer in men, with over 1.4 million newly diagnosed cases in 2020 (Sung et al., 2021). Due to its highly heterogenous nature, the course of the disease tends to vary between patients, with some developing aggressive forms of cancer with a high risk of metastasis and some having a slowly progressing disease without the need for active treatment (Flores-Téllez and Baena, 2022). Currently, diagnostic examinations include the measurement of PSA levels in the blood, digital rectal examination, and histological analysis of transrectal ultrasound scan (TRUS)-guided biopsies (Descotes, 2019; Lomas and Ahmed, 2020). However, these tests are known to pose some detrimental issues - PSA tests have low specificity and are unable to differentiate PCa from benign prostate hyperplasia (BPH), whereas histological analysis of biopsy material tends to be subjective and has a high risk of infection at the puncture site (Loeb et al., 2013; Ahmed et al., 2017; Ilic et al., 2018). Furthermore, a new problem has arisen in recent decades - overdiagnosis and overtreatment of PCa, since the current diagnostic approaches cannot reliably differentiate between fast-progressing cancer requiring aggressive treatment and indolent forms of the disease that can be managed by active surveillance (Loeb et al., 2014). Therefore, more precise and less invasive tools for the detection and monitoring of PCa still represent unmet clinical needs in the management of PCa.

In recent years, liquid biopsies have emerged as a popular, non-invasive alternative to tissue biopsies. The term refers to the analysis of cancer-derived molecules in any biofluid to gain information about a potential or existing malignancy (Lu et al., 2019). Recently, extracellular vesicles (EVs) have gained distinct recognition as a potential source of biomarkers in PCa patients’ blood and urine (Hessvik et al., 2013; Li et al., 2014; Ramirez-Garrastacho et al., 2022a). EVs are heterogenous membrane-bound particles with diverse roles in cellular communication and biomolecule transport that are secreted by virtually all cell types into the extracellular space (Yáñez-Mó et al., 2015; van Niel et al., 2018). They contain proteins, lipids, nucleic acids, and various metabolites, depicting the contents of cells from which they originate (Abels and Breakefield, 2016). Furthermore, cancer-derived EVs have been shown to contain altered cargo and play a significant role in tumor development, proliferation, metastasis, and resistance to treatment (Cocks et al., 2021; Hell et al., 2021). Several studies have been carried out identifying specific PCa RNA biomarkers in plasma and urinary EVs (Nilsson et al., 2009; Samsonov et al., 2016; Foj et al., 2017; Matsuzaki et al., 2021), however, only a few of them have been validated in independent studies (Yu et al., 2021; Ramirez-Garrastacho et al., 2022a).

In a previous study, we carried out RNA sequencing analysis of plasma and urinary EVs collected before and after radical prostatectomy, and matched tumor and normal prostate tissues from 10 PCa patients to identify PCa-derived RNA biomarkers (Bajo-Santos et al., 2023). We hypothesized that the levels of PCa-derived RNA biomarkers would decrease in post-operation samples compared to pre-operation samples if a substantial fraction of the given RNA in the given biofluid comes from PCa and/or prostate, therefore we searched for EV RNAs that are overexpressed in tumor tissues, present in the pre-operation EVs above the set threshold and decrease after prostatectomy. In the current study, we aimed to validate the biomarker candidates by RT-ddPCR in independent cohorts of plasma and urine samples from PCa and BPH patients and establish their diagnostic values.

## Materials and methods

### Patient recruitment and sample collection

A total of 52 patients diagnosed with PCa and 51 patients diagnosed with BPH were enrolled in this study at Riga East University Hospital between October 2018 and January 2020. The patients were followed up until September 2021. The inclusion criteria for PCa patients were as follows: (1) recent diagnosis of resectable PCa confirmed through histopathological analysis TRUS-guided biopsy, (2) scheduled prostatectomy, and (3) age over 18. PCa patients were excluded if they had: (1) other concurrent oncological conditions, (2) undergone chemotherapy, radiation, or hormonal treatment prior to the study, (3) urinary tract infection during sample collection, (4) received a blood transfusion within the past 4 months, (5) long-term catheter use, or (6) inability to provide informed consent. The inclusion criteria for BPH patients were: (1) recent diagnosis of BPH confirmed through histopathological analysis TRUS-guided biopsy and (2) blood PSA level >2.5 ng/mL and <50 ng/mL. Further clinical characteristics of the patients included in this study are provided in Table 1.

**TABLE 1:** Patient characteristics.

		Validation cohort		Test cohort				Validation cohort			
		PCa pre-Op vs Post-OP		PCa		BPH		PCa		BPH	
Number		30		20		20		31		31	
Age (Median, years)		66		66.5		67		64		64	
Age (range)		49-77		49-74		53-84		50-88		45-80	
Age (*p*-value^a^)				0.65				0.46			
Diagnostic PSA (ng/mL)		Number	%	Number	%	Number	%	Number	%	Number	%
	<4	2	6.67	2	10	2	10	1	3.2	11	35.5
	4-10	17	56.67	8	40	9	45	18	58.1	14	45.2
	>10	11	36.67	10	50	9	45	12	38.7	6	19.4
Gleason score											
	6	12	40	7	35	NA **	NA	11	35.5	NA	NA
	7 (3 + 4)	3	10	3	15	NA	NA	5	16.1	NA	NA
	7 (4 + 3)	9	30	4	20	NA	NA	14	45.2	NA	NA
	8	3	10	3	15	NA	NA	1	3.2	NA	NA
	9	3	10	3	15	NA	NA	-	-	NA	NA

^a^ Mann-Whitney test; **NA, Not Applicable.

Patient samples were collected at two time points: before and 3–4 months after radical prostatectomy, denoted as Pre-Op and Post-Op, respectively. Approximately 60 mL of the first morning urine were collected from each patient and centrifuged at 2000 *g* for 15 min at room temperature. The resulting aliquots were stored at −80°C. Blood samples were collected in EDTA-coated tubes and processed at room temperature within 2 h after the blood draw. A two-step plasma isolation process was performed on the blood samples by 2x centrifugation at 3000 *g* for 10 min at room temperature. The plasma samples were aliquoted and stored at −80°C.

A uropathologist obtained tumor and normal tissue samples immediately following radical prostatectomy. One portion of the tissue samples underwent a histopathological evaluation to determine the presence or absence of tumor cells and to assess the Gleason score. The remaining portion was preserved in RNALater solution (QIAGEN) and stored at −20°C.

The study was conducted in accordance with the principles outlined in the Declaration of Helsinki. The clinical samples and information was collected after obtaining informed written consent from the patients, ensuring their anonymity. The study protocol was approved by the Latvian Central Medical Ethics Committee (decision No. 01-29.1/488).

### Isolation and characterization of extracellular vesicles

EVs were isolated from patient plasma and urine samples using size exclusion chromatography (SEC). In a water bath, frozen urine (20 mL) and plasma (1 mL) samples were thawed at +37°C. Urine samples were centrifuged at 10 000 *g* for 15 min at +4°C to remove larger debris and uromodulin and concentrated up to 1 mL using 100 kDa centrifugal filters (Merck Millipore, USA). Following this, urine and plasma samples were loaded onto Sepharose CL2B 10 mL columns and the eluate was collected in 15 0.5 mL fractions. Each fraction was measured with Zetasizer Nano ZS (Malvern, UK) and fractions containing at least 70% of particles larger than 30 nm in diameter were combined and concentrated up to 100 µL using 3 kDa centrifugal filters (Merck Millipore, USA). EV samples were treated with Proteinase K (1 mg/mL) (ThermoFisher Scientific, USA) for 60 min at RT followed by treatment with RNAse A (100 ng/μL) (ThermoFisher Scientific, USA) treatment for 15 min at RT. Nanoparticle tracking analysis (NTA) using Nanosight NS500 instrument (Malvern, UK) was performed on each sample to determine the approximate size and concentration of EVs as described before (Endzeliņš et al., 2017).

### RNA isolation

RNA was extracted from EV samples using miRNeasy Micro Kit (Qiagen, Germany) following the manufacturer’s protocol. Additionally, DNase treatment was performed on the column according to the manufacturer’s instructions. The concentration of EV-RNA was measured using Agilent 2100 Bioanalyzer and RNA 6000 Pico Kit (Agilent Technologies, USA).

### Reverse transcription - droplet digital PCR

cDNA was synthesized using miRCURY LNA RT kit according to the manufacturer’s instructions (Qiagen, Germany). Nine µl (half of the entire yield) of RNA was used for cDNA synthesis and further diluted 1:2 in nuclease-free water (ThermoFisher Scientific, USA). Each droplet digital PCR (ddPCR) reaction containing 2 µL of diluted cDNA, 10 µL of 2x QX200 ddPCR EvaGreen Supermix (Bio-Rad, USA), 7 µL of nuclease-free water (ThermoFisher Scientific, USA) and 1 µL of primer mix (Qiagen, Germany (Supplementary Table S1) was loaded onto a DG8 cartridge (Bio-Rad, USA). Then, 70 µL of QX200 Droplet Generation Oil for EvaGreen (Bio-Rad, USA) were added to the cartridge and a DG8 gasket (Bio-Rad, USA) was hooked over the cartridge, followed by the insertion of the cartridge into a QX200 Droplet Generator (Bio-Rad, USA). 40 μL of the generated droplets were then loaded onto a clear 96-well semi-skirted PCR plate (Bio-Rad, USA) and the plate was heat sealed with a pierceable foil (Bio-Rad, USA) using ALPS 25 manual heat sealer (ThermoFisher Scientific, USA). The PCR reaction was carried out using a T100 Thermal Cycler (Bio-Rad, USA) under the following conditions: 95°C for 5 min; 40 cycles at 95°C for 30 s followed by specific primer annealing temperature (Supplementary Table S1); 4°C for 5 min; 90°C for 5 min and indefinite hold at 4°C. The program was run at a 2°C/sec rampage rate. After PCR, the plate was allowed to cool for at least 2 h at +4°C and then was read using a QX200 Droplet Reader (Bio-Rad, USA). Results were analyzed using QuantaSoft Software (Bio-Rad, USA). Before performing RT-ddPCR experiments, the optimal annealing temperature for each primer pair was determined by running PCR reactions on a temperature gradient (50°C-60°C).

### Statistical analysis

Statistical analyses were performed using GraphPad Prism 9.0 (GraphPad, USA) and RStudio 4.2 (RStudio Team, USA). Comparison between PCa Pre-Op vs*.* Post-Op data was assessed using Wilcoxon matched-pairs signed rank test. Mann-Whitney test was used for comparing the biomarker levels in independent groups. RNA biomarker models were constructed with glm package (Venables and Ripley, 2002) by fitting generalized linear models based on Gaussian identity function. To assess NKX3-1 expression level in the datasets available at The Cancer Genome Atlas (TCGA), normalized gene expression values (TPM) were obtained from the Gene Expression Omnibus (accession number GSE62944) (Rahman et al., 2015). We selected the Prostate Adenocarcinoma (PRAD) NKX3-1 subset of data and tested it by Mann–Whitney test.

## Results

### Extracellular vesicle isolation and quality control

**FIGURE 1:**
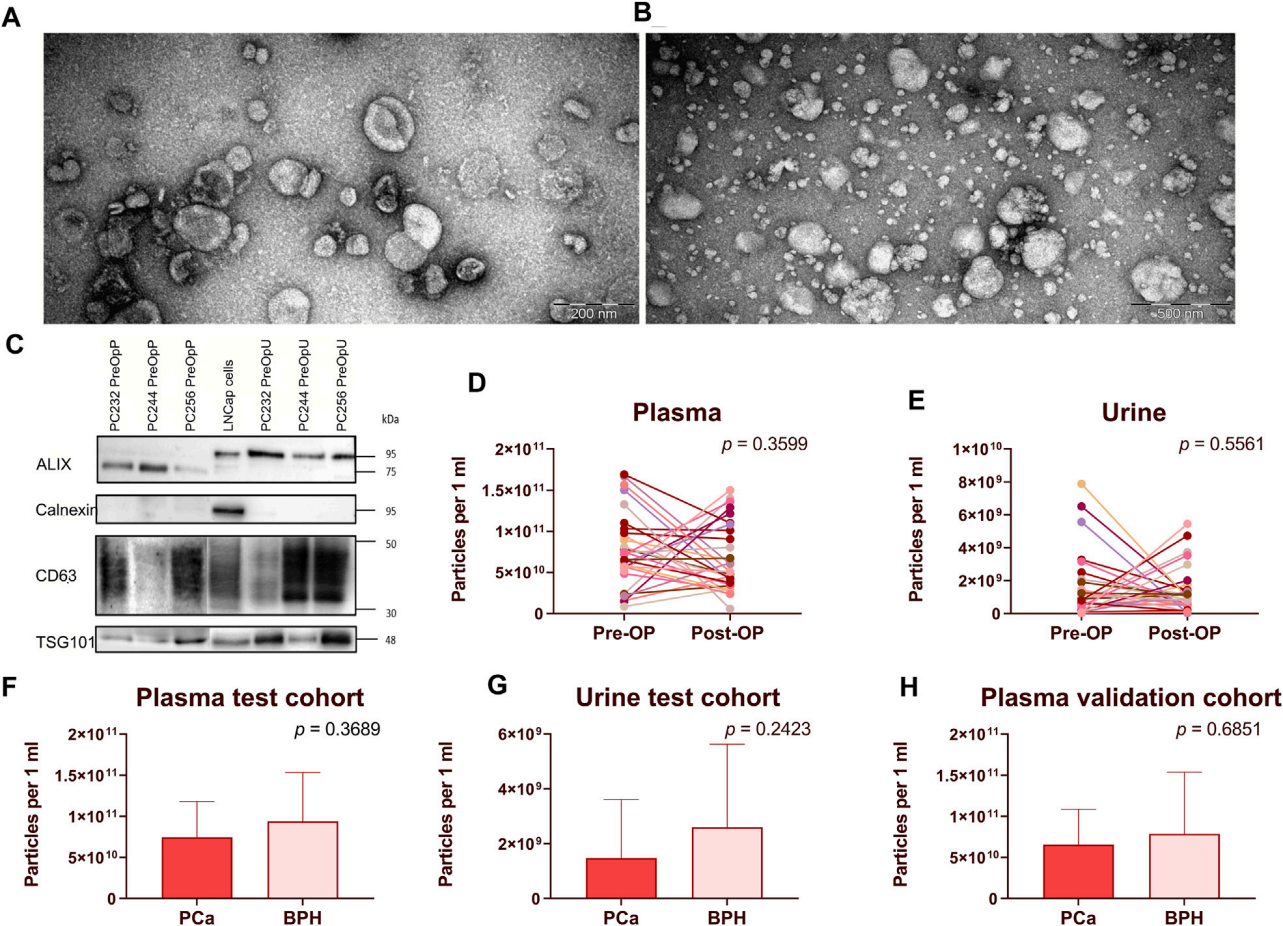
EV isolation and quality control. **(A,B)** Transmission electron microscopy images showing EVs isolated from urine **(A)** and plasma **(B)**. Western blot analysis of ALIX, Calnexin, CD63 and TSG101 in plasma and urinary EVs from 3 randomly selected patients as well as LNCap prostate cancer cells **(C)**. Paired dot plots showing EV concentration per ml of plasma **(D)** and urine **(E)** samples before and after radical prostatectomy. Wilcoxon matched-pairs signed rank test was used to assess the statistical significance of the differences between groups. **(F–H)** Box plots showing EV concentration per ml of plasma **(F–H)** and urine **(G)** in patients with PCa and BPH. Figures 1F, 1G represent patients in the test cohort and Figure 1H represents patients in the validation cohort. Mann-Whitney test was used to assess the statistical significance of the differences between groups. *p*-value < 0.05 was considered significant.

EVs were isolated from PCa and BPH patients’ plasma and urine samples following a previously established protocol (Endzeliņš et al., 2017). All samples were categorized into the following groups: (1) RNASeq validation cohort (30 PCa Pre-Op vs Post-Op; plasma and urine), aimed to validate the results of the previous EV-RNA sequencing study (Bajo-Santos et al., 2023); (2) test cohort (20 PCa Pre-Op vs 20 BPH; plasma and urine), aimed to assess potential RNA biomarker ability to discriminate between PCa and BPH; (3) validation cohort (31 PCa Pre-Op vs 31 BPH, plasma), aimed to confirm RNA biomarker diagnostic value. The quality of the obtained EV preps was checked by transmission electron microscopy for urine (Figure 1A) and plasma (Figure 1B) as well as Western blot analysis with antibodies against typical EV markers ALIX, TSG101 and CD63, and calnexin as a negative control of plasma and urinary EVs from randomly selected 3 patients (Figure 1C) and the results are published before (Bajo-Santos et al., 2023). Routinely, EV yields in all samples were assessed by NTA. Results show that the number of particles ranges from 8.60 × 10^9^ to 1.67 × 10^11^ in Pre-Op and 5.6 × 10^9^ to 1.50 × 10^11^ particles per ml of plasma in Post-Op plasma samples in the RNAseq validation cohort (Figure 1D) and 2.60 × 10^7^ to 7.89 × 10^9^ in Pre-Op and 3.19 × 10^7^ to 5.45 × 10^9^ particles per ml of urine in Post-Op urine samples (Figure 1E). In the test cohort, EV numbers ranged from 8.6 × 10^9^ to 1.67 × 10^11^ in PCa and 7.62 × 10^9^ to 1.93 × 10^11^ in BPH plasma samples (Figure 1F) and 2.6 × 10^7^ to 6.51 × 10^9^ in PCa and 1.94 × 10^7^ to 8.97 × 10^9^ in BPH urine samples (Figure 1G). In the validation cohort, EV concentration per ml ranged from 1.57 × 10^10^ to 1.16 × 10^11^ in PCa and 1.69 × 10^10^ to 4.05 × 10^11^ in BPH plasma samples (Figure 1H). No statistically significant differences between the groups of samples were found.

### Biomarker candidates

A total of 11 potential RNA biomarker candidates representing various RNA biotypes were selected for this study based on their overexpression in tumor tissues, relatively high levels in PreOp EVs and decreased levels in PostOp EVs in our previous RNA sequencing study (Bajo-Santos et al., 2023). Additionally, 4 RNA biomarker candidates were included (PCA3, PCAT14, tRNA-Phe-GAA-1-1/2/3/4/5/6-3b (tRF-Phe-GAA-3b) and tRNA-Lys-CTT-(1-1/2)/(4-1)-5c (tRF-Lys-CTT-5c)) based on previously reported diagnostic and/or prognostic values. Further information on each of the RNA candidates is provided in Table 2. For each of the candidates, LNA-based primers were designed (utilizing either QuantiNova LNA PCR custom assays or miRCURY LNA miRNA PCR assays, depending on the target length and RNA biotype), employing the GeneGlobe platform from Qiagen, Germany. PCR assay numbers are provided in Supplementary Table S1.

**TABLE 2:** Selected biomarker candidates.

Gene name	PCa vs normal tissues		PreOp vs PostOp plasma		PreOp vs PostOp urine		Published data supporting biomarker validity
	Log2FC	adj. *p*-value	Log2FC	adj. *p*-value	Log2FC	adj. *p*-value	
mRNA							
GLO1	1.47	3.16 × 10^−13^	0	0	4.07	3.98 × 10^−3^	Rounds et al. (2021)
NKX3-1	2.76	4.67 × 10^−5^	0.76	0.97	2.67	0.012	Huang et al. (2018)
AMD1	2.00	2.78 × 10^−22^	−0.55	0.97	3.97	1.11 × 10^−3^	Bajo-Santos et al. (2023)
PMEPA1	2.85	5.35 × 10^−16^	−0.42	0.97	2.72	2.03 × 10^−3^	Sharad et al. (2020)
RBM47	2.74	1.36 × 10^−8^	1.37	0.97	2.48	9.17 × 10^−3^	Bajo-Santos et al. (2023)
MAZ	3.37	1.63 × 10^−20^	2.75	0.97	3.54	1.3 × 10^−4^	Bajo-Santos et al. (2023)
**lncRNA**							
PCA3	9.08	1.32 × 10^−85^	−0.24	0.96	1.88	0.36	Woo et al. (2019)
PCAT14	7.17	129 × 10^−32^	0	0	2.45	0.34	Yan et al. (2021)
**tRFs**							
tRF-Phe-GAA-3b	−0.59	0.92	−2.00	0.43	1.19	0.62	Olvedy et al. (2016)
tRF-Lys-CTT-5c	−0.08	0.92	1.4	0.43	0.94	0.62	Olvedy et al. (2016)
**miRNAs**							
miR-375-3p	2.90	1.51 × 10^−4^	1.01	0.99	1.45	3.22 × 10^−3^	Foj et al. (2017)
miR-92a -1-5p	1.73	7.95 × 10^−4^	0.68	0.99	1.90	3.22 × 10^−3^	Mercadal et al. (2020)
miR-27a - 5p	1.06	2.80 × 10^−4^	−0.54	0.99	0.63	0.62	Ku et al. (2021)
miR-196a-5p	1.13	6.51 × 10^−5^	0.27	0.99	0.57	0.56	Rodríguez et al. (2017)
**piRNA**							
piR-28004	12.30	0	−4.88	0.23	6.47	1.77 × 10^−3^	Bajo-Santos et al. (2023)

### Normalization strategies

Given the present lack of established PCR data normalization methodologies for the analysis of urinary EVs, we adopted a triad of distinct normalization strategies. The first approach involved data normalization relative to 1 mL of the biofluid from which the EVs were extracted. The second strategy entailed data normalization against the total EV count, as quantified through NTA. The third method encompassed data normalization with respect to two candidate internal control miRNAs, namely, let-7f-5p and miR-26a-5p, as discerned in a preceding sequencing study (Bajo-Santos et al., 2023) and published by others (Tonge and Gant, 2013; Gouin et al., 2017). These miRNAs were detected across all EV samples and exhibited minimal variance, thereby facilitating the reduction of data variability in pursuit of enhanced consistency. These strategies were devised with the primary objective of minimizing data variance within our dataset.

For each normalization variant, variation coefficients for each RNA biomarker were calculated from the raw PCR data as shown in Table 3. It was concluded that on average, normalizing data against 1 mL of biofluid from which EVs were isolated showed the least variation among the datasets. All further analyses were conducted based on this normalization method.

**TABLE 3:** Variation coefficients of RT-ddPCR results in plasma and urinary EV samples under different normalization strategies.

Marker name	Plasma			Urine		
	Volume (1 mL)	Internal control miRNA	EV count	Volume (1 mL)	Internal control miRNA	EV count
GLO1	1.31	2.39	2.11	1.50	2.97	1.71
NKX3-1	1.11	2.48	1.79	1.70	2.79	2.37
AMD1	1.43	3.11	1.91	1.66	2.94	2.02
PMEPA1	1.29	2.77	1.49	1.33	3.53	2.46
RBM47	1.44	3.12	1.51	1.40	2.73	2.31
MAZ	1.20	2.98	1.58	1.51	3.41	2.09
PCA3	1.29	3.25	1.65	1.22	2.55	2.58
PCAT14	1.10	2.87	1.60	1.47	3.74	1.99
tRF-Phe-GAA-3b	1.43	2.56	1.52	1.65	2.16	1.42
tRF-Lys-CTT-5c	1.42	2.33	1.59	1.89	1.54	1.20
miR-375	2.51	3.94	2.18	2.10	1.73	1.89
miR-92a	2.10	3.17	2.75	2.01	2.74	2.40
miR-27a	0.60	2.73	1.29	0.75	2.75	2.20
miR-196	1.79	3.28	2.55	2.39	1.17	1.47
piR-28004	1.07	1.54	1.53	1.39	1.27	1.32
**Average**	1.41	2.83	1.80	1.60	2.53	2.01

### Comparison of RNA biomarker levels in PCa pre-Op versus post-Op EVs

To validate the results of the previous RNA sequencing study and identify biomarkers, whose main tissue source in plasma or urinary EVs was PCa and/or normal prostate, we compared their levels in plasma and urinary EV samples collected before and after radical prostatectomy from 30 PCa patients.

**FIGURE 2:**
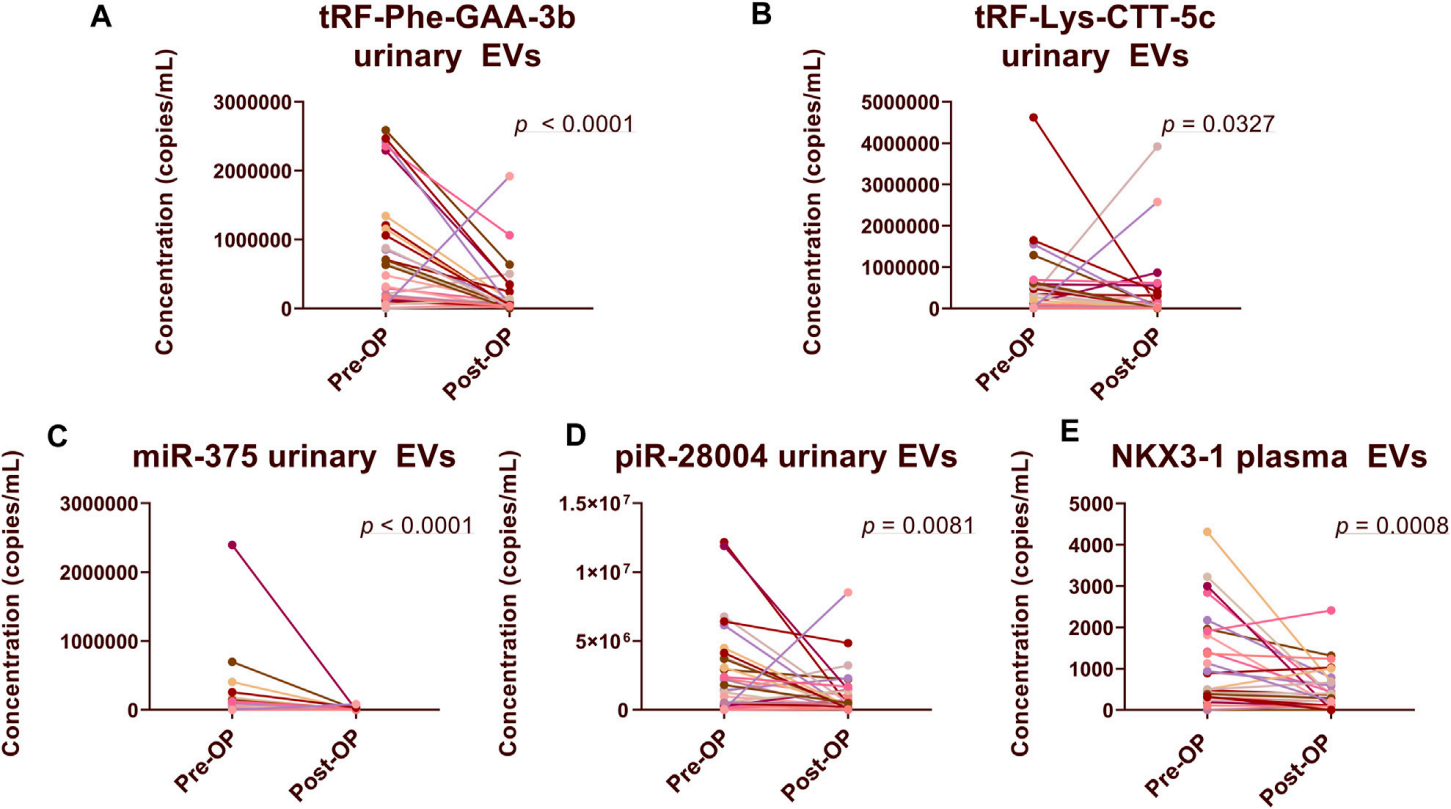
Comparison of RNA biomarker levels in Pre-Op vs*.* Post-Op plasma and urinary EVs. Paired dot plots show copy numbers of RNA biomarkers per ml of urine **(A–D)** and plasma **(E)** collected before and after radical prostatectomy in 30 PCa patients. Wilcoxon matched-pairs signed rank test was used to assess the statistical significance of the differences between groups. *p*-value < 0.05 was considered significant.

In urinary EVs, the levels of four biomarker candidates: tRF-Phe-GAA-3b (FC = 3.73; *p* < 0.0001), tRF-Lys-CTT-5c (FC = 1.46, *p* = 0.0327), miR-375-3p (FC = 14.85, *p* < 0.0001) and piR-28004 (FC = 2.45, *p* = 0.0081) were significantly decreased following radical prostatectomy (Figures 2A–D), which is in concordance to our previous data. However, in plasma EVs, the levels of only one biomarker candidate—NKX3-1 (FC = 2.32, *p* = 0.0008) significantly decreased after prostatectomy (Figure 2E). The levels of several other biomarker candidates in plasma (Supplementary Image S1) and urinary (Supplementary Image S2) EVs tended to decrease following prostatectomy yet did not reach statistical significance.

### Diagnostic performance of RNA biomarker candidates

**FIGURE 3:**
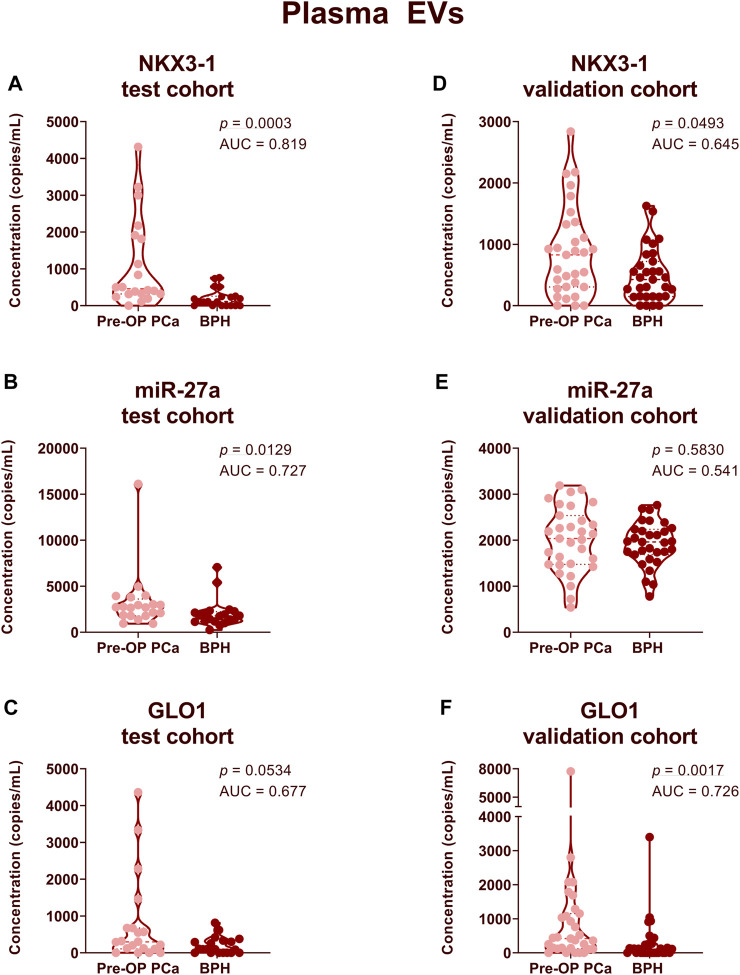
Comparison of RNA biomarker levels in patients with PCa vs BPH in plasma EV samples. Violin plots show copy numbers of RNA biomarkers NKX3-1 **(A,D)**, miR-27a **(B,E)** and GLO1 **(C,F)** per ml of plasma in the test cohort (20 PCa, 20 BPH) **(A–C)** and validation cohort (31 PCa, 31 BPH) **(D–F)**. Mann-Whitney test was used to assess the statistical significance of the differences between groups. *p*-value < 0.05 was considered significant.

In order to assess whether the selected RNA biomarker candidates could distinguish between patients with PCa and BPH, we compared their levels in a test cohort of 20 PCa and 20 BPH patient plasma and urinary EV samples. In plasma EV samples, the levels of NKX3-1 (FC = 5.42, *p* = 0.0003, AUC = 0.82) and miR-27a (FC = 1.61, *p* = 0.0129, AUC = 0.73) were significantly higher in PCa patients (Figures 3A,B). The levels of GLO1 (FC = 3.99, *p* = 0.0534, AUC = 0.68) were also higher in PCa patients, but the difference was not statistically significant (Figure 3C). The levels of several other biomarker candidates such as PCAT14 and PMEPA1 were elevated in PCa plasma EVs yet failed to reach statistical significance (Supplementary Image S3). None of the potential RNA candidates reached statistical significance between the two groups in urinary EVs (Supplementary Image S4).

Next, we aimed to validate these results in an independent validation cohort of 31 PCa and 31 BPH patient plasma samples. At this point, we did not include urinary EV samples as they showed significantly lower diagnostic potential compared to plasma EVs. The level of NKX3-1 remained significantly higher in PCa patients compared to BPH patients (FC = 1.76, *p* = 0.0493, AUC = 0.65) (Figure 3D). However, miR-27a failed to discriminate between PCa and BPH in an independent cohort (Figure 3E). In an independent patient cohort, the levels of GLO1 were significantly higher in PCa patients compared to BPH patients (FC = 3.10, *p* = 0.0017, AUC = 0.73) (Figure 3F).

**FIGURE 4:**
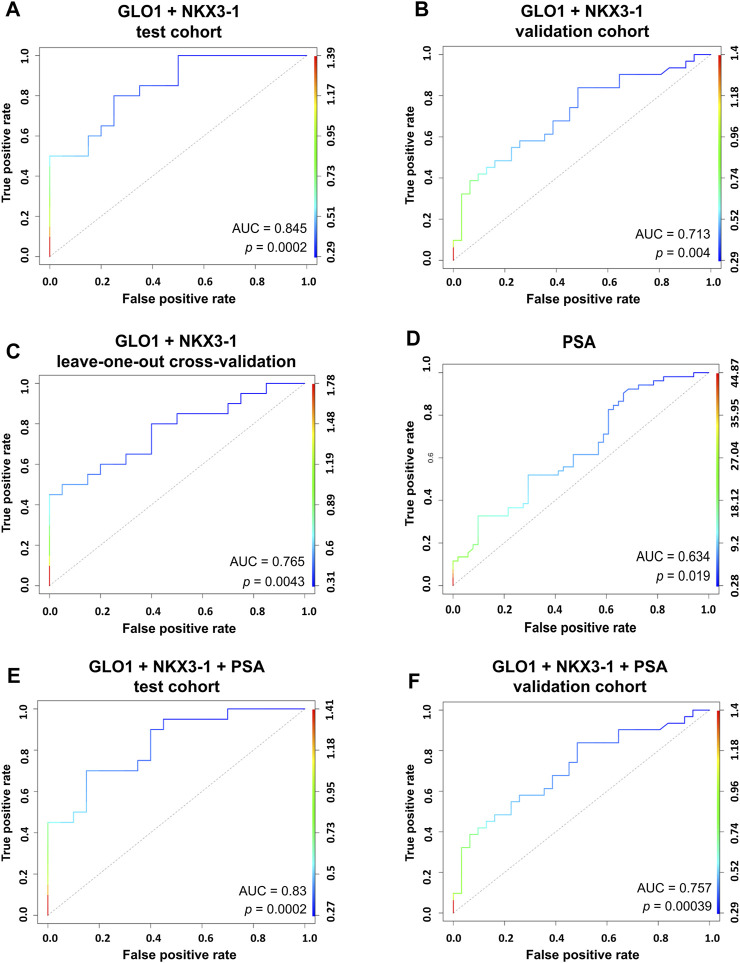
RNA biomarker models. Generalized linear models combining the top two performing candidates NKX3-1 and GLO1 based on Gaussian identity function show an ability to discriminate between PCa and BPH in a test cohort of 20 PCa vs 20 BPH patient plasma EVs **(A)** and a validation cohort of 31 PCa vs 31 BPH patient plasma EVs **(B)**. Leave-one-out cross-validation of the model **(C)**. ROC curve for the PSA test **(D)**. A combined model of PSA measurements and two RNA biomarkers (NKX3-1 and GLO1) showing the ability to discriminate between PCa and BPH in a test cohort of 20 PCa vs 20 BPH patient plasma EVs **(E)** and a validation cohort of 31 PCa vs 31 BPH patient plasma EVs **(F)**.

Next, we combined the two top-performing candidates - NKX3-1 and GLO1—in a biomarker model based on Gaussian identity function. The two-biomarker model showed an AUC of 0.845 (Figure 4A) in the test cohort and an AUC of 0.713 in the validation cohort (Figure 4B). The leave-one-out cross-validation of the model yielded an AUC value of 0.765, thus further confirming its viability (Figure 4C). PSA test showed a poor diagnostic value in our cohort of patients as it could distinguish PCa from BPH with an AUC of 0.634. Adding PSA to the two-biomarker model did not improve the performance of the model in the test cohort (Figure 4E), whereas it slightly increased its diagnostic performance in the validation set by increasing the AUC from 0.713 to 0.757 (Figure 4F).

### Prognostic performance of RNA biomarker candidates

**FIGURE 5:**
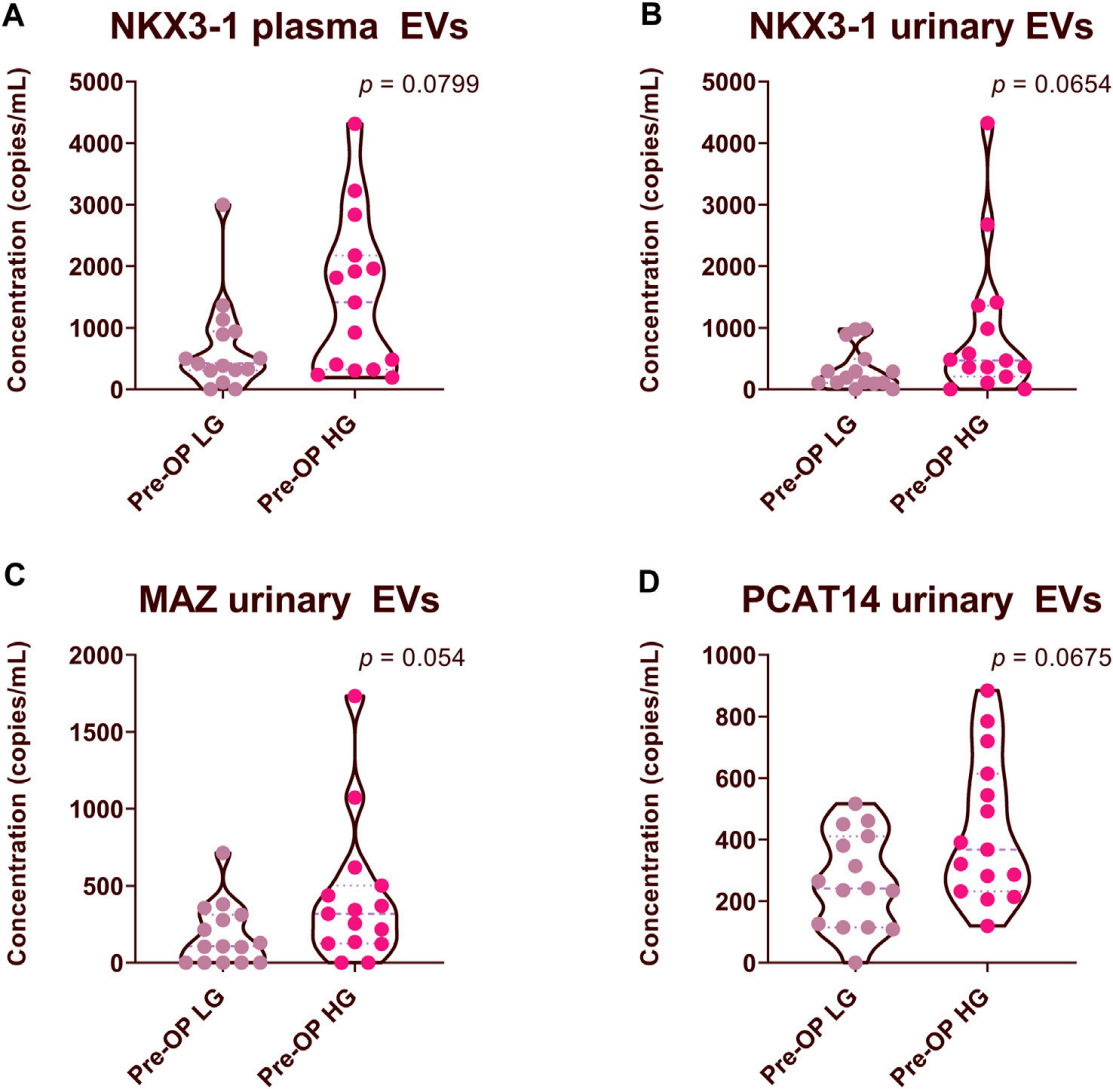
Comparison of RNA biomarker levels in patients with high Gleason (HG) and low Gleason (LG) scores. Violin plots show copy numbers of RNA biomarkers per 1 mL of patient plasma **(A)** and urinary EVs **(B–D)**. Mann-Whitney test was used to assess the statistical significance of the differences between groups. *p*-value < 0.05 was considered significant.

To assess whether the RNA biomarker candidates have the ability to discriminate between low and high-grade prostate cancer, we divided the data from 30 PCa Pre-Op plasma and urinary EV samples, into two groups: high Gleason (HG) (Gleason score 4 + 3, 4 + 5 and 4 + 5) and low Gleason (LG) (Gleason score 3 + 3 and 3 + 4) groups, comprising of 15 patients in each group. We found that the levels of NKX3-1 both in plasma and urinary EVs tended to be higher in patients with HG group, yet did not reach statistical significance (Figures 5A, B). Similarly, the levels of MAZ and PCAT14 in urinary EVs tended to be higher in patients with a high Gleason score (Figures 5C,D).

### Expression levels and mutational analysis of NKX3-1 in PCa

**FIGURE 6:**
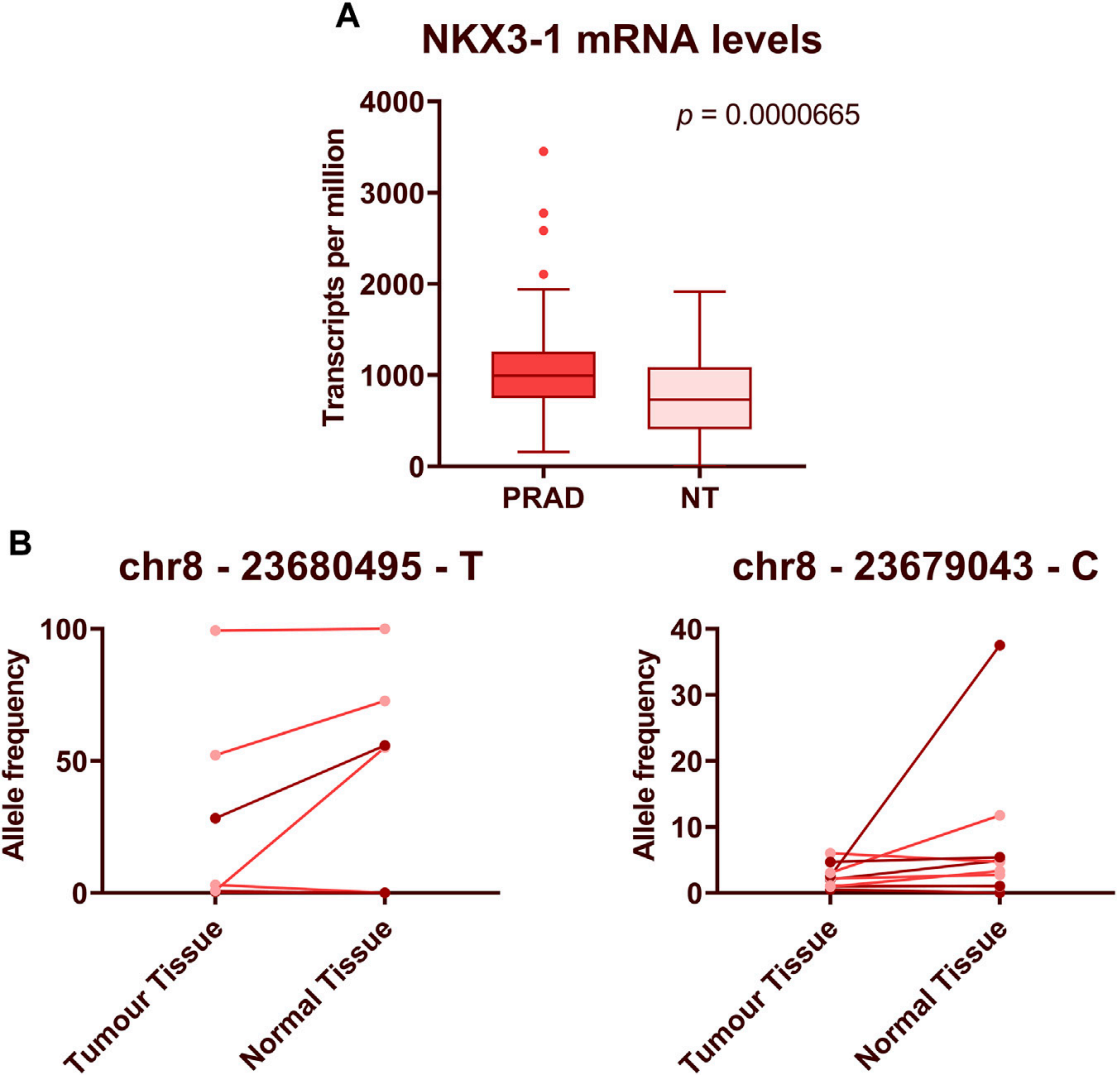
Expression levels and mutational analysis of NKX3-1 in PCa tissues. **(A)** The box plot shows NKX3-1 mRNA levels in prostate adenocarcinoma (PRAD) and normal prostate tissue (NT) gene expression datasets from TCGA database. Mann-Whitney test was used to assess the statistical significance of the differences between groups. *p*-value < 0.05 was considered significant. **(B)** Paired dot plots show allele frequencies of 2 SNPs obtained from RNA sequencing data in tumor and normal prostate tissues from 10 PCa patients.

*NKX3-1* gene encodes a transcription factor that functions as a prostate-specific tumor suppressor and its protein levels are commonly decreased in PCa (Padmanabhan et al., 2016). We have found that its mRNA is overexpressed in PCa as compared to adjacent normal prostate tissues (Bajo-Santos et al., 2023) and the levels of NKX3-1 mRNA are significantly higher in plasma EVs from patients with PCa than BPH, and higher levels tend to associate with higher Gleason score that seems to contradict with its tumor suppressor’s role in PCa. To gain a comprehensive view of its expression level in PCa, we analyzed the transcriptomic data available at The Cancer Genome Atlas Program (TCGA). Comparison of NKX3-1 mRNA levels in RNAseq data from 502 prostate adenocarcinoma cases and 52 normal prostate tissues showed that its level in PCa tissues is substantially higher than in normal prostate tissues (Mann-Whitney test, *p* = 6.65 × 10^−5^) (Figure 6A). To search for somatic mutations in NKX3-1 gene, we compared the RNA sequences obtained from PCa and normal prostate tissues in our previous study (Bajo-Santos et al., 2023). We were unable to identify any somatic point mutations in the tumor tissues, whereas the frequencies of several common SNPs suggested that one allele of NKX3-1 is lost in the tumor tissues of 4 out of 10 PCa patients (Figure 6B).

## Discussion

Liquid biopsies have emerged as an increasingly favored alternative to traditional tissue biopsies for cancer diagnosis and surveillance. Biological fluids like plasma and urine hold rich reservoirs of cancer-related information, encompassing circulating cancer cells, cell-free DNA and RNA, diverse proteins, and EVs (Lu et al., 2019; Ramirez-Garrastacho et al., 2022a). Previously deemed metabolic byproducts, EVs have been revealed to harbor an extensive array of metabolites and nucleic acids derived from their parent cells, assuming pivotal roles in intercellular communication, cancer progression, and metastasis (Doyle and Wang, 2019).

Several studies, including our recent study of breast cancer, have shown that EV concentrations in body fluids are increased in cancer patients as compared to healthy controls and their levels reflect clinical events such as chemotherapy or surgery, thus suggesting that the excess EVs are produced in the body due to the disease process or treatment (Lázaro-Ibáñez et al., 2014; Cappello et al., 2017; Krafft et al., 2017; Menck et al., 2017; König et al., 2018; Sadovska et al., 2022). Therefore, we expected that the EV numbers should decrease after the surgical removal of the tumor. However, our data did not reveal a statistically significant reduction in EV yields in patients undergoing radical prostatectomy. This suggests that the prostate and/or PCa does not produce sufficiently large amounts of EVs that could substantially influence the overall EV counts in the body. This could be attributed to the relatively small size of the prostate gland or to lower EV secretion rates. Of note, we observed a tendency towards elevated EV concentrations in patients with BPH compared to those with PCa. While these differences did not reach statistical significance, the observations align with findings from a prior study suggesting that the enlarged prostate size observed in BPH patients may contribute to an overall increase in EV counts (Salvi et al., 2021). On the other hand, it can not be excluded that the controversial findings regarding the EV counts are related to technical issues such as co-isolation of lipoprotein particles that could potentially introduce distortions to NTA measurements, differences in the EV isolation and counting methods and pre-analytical variables (Ramirez-Garrastacho et al., 2022a).

The primary goal of this study was to validate RNA biomarker candidates identified in our previous sequencing study (Bajo-Santos et al., 2023) and develop clinically applicable ddPCR-based assays for the detection and monitoring of PCa. We utilized RT-ddPCR, commonly employed for the absolute quantification of nucleic acids. This method has gained significant traction in oncology, finding application in various areas such as absolute allele quantification, detection of rare mutations, assessment of copy number variations, and DNA methylation analysis. Key benefits include its absolute quantification, high specificity, and high sensitivity, facilitating the precise analysis of minute copy numbers within a sample without a need for standard curve construction. Its capacity to detect and accurately quantify low copy numbers of target molecules aligns seamlessly with EV-enclosed cancer biomarker analysis requirements in liquid biopsies (Palacín-Aliana et al., 2021). The main technical challenges we encountered during this study were related to the fragmented nature of EV-enclosed RNAs and the normalization of PCR data.

The greatest part of the biomarker candidates identified in our EV RNA sequencing study were mRNAs. Alignment of EV RNA reads against the reference sequences clearly showed that the EV RNA is fragmented, however, it was not clear if the fragmentation is entirely random or there are some preferential cleavage sites or sorting mechanisms resulting in the enrichment of some specific fragments in EVs. This represents a challenge in the PCR primer design. Although we succeeded in developing several successful PCR assays for the amplification of mRNA fragments, currently there are no universal rules for selecting appropriate target sequences.

The foremost hurdle, however, lies in the normalization of PCR data. In the RNA sequencing analysis, gene expression values can be reliably normalized using global normalization approaches (X. Li et al., 2020) allowing accurate comparison of the gene expression levels across samples, however, these approaches can not be transferred to the PCR results and currently no robust normalization approach exists for the analysis of PCR results obtained from extracellular RNAs (Erdbrügger et al., 2021). We explored three data normalization strategies to identify an approach that minimized dataset variations. Ultimately, we determined that normalizing data to the volume of the analyzed biofluids yielded the least variable results. This normalization technique appears to be effective for plasma samples, given that its volume is generally less susceptible to variability, a trend documented in earlier studies (Endzeliņš et al., 2017; Royo et al., 2020). However, urine demonstrates greater heterogeneity in terms of composition and concentration, influenced by diverse factors including patient diet, health, and lifestyle, therefore such an approach is not optimal for urinary EVs. Although several alternative normalization approaches for urinary EVs have been proposed, currently none of them is widely accepted in the EV research community and finding a robust normalization approach is still an unmet need (Blijdorp et al., 2021; Erdbrügger et al., 2021).

Furthermore, only a minor fraction of the total EVs in biofluids appears to be derived from the prostate, rendering the detection of prostate-specific RNA fragments exceedingly challenging. A potential avenue for addressing this challenge is urine collection subsequent to a digital rectal examination, as such prostate stimulation has been demonstrated to elevate prostate-specific EV counts in urine (Hendriks et al., 2016; Pellegrini et al., 2017). Additionally, a lack of standardized methodologies for EV isolation and the preservation of biological fluids underscores another predicament. This lack of consensus contributes to remarkable variation across EV research studies, often leading to difficulties in replicating or validating results.

Urine is the most frequently employed biofluid in liquid biopsies of PCa. Its merits encompass non-invasive collection, the capacity to collect and conserve substantial volumes simultaneously, and a comparatively limited count of contributing organs for urinary EVs (Ramirez-Garrastacho et al., 2022a). It is a preferential biofluid for the diagnosis and active surveillance of PCa, whereas blood is likely to be the most suitable biofluid for the post-operative monitoring of PCa patients (Ramirez-Garrastacho et al., 2022a). In this study, we used blood plasma instead of serum. This decision was motivated by the observation that during the processing of serum, EVs from platelets tend to co-isolate, thereby augmenting the total EV count and subsequently introducing additional variability (Coumans et al., 2017; Campos-Fernández et al., 2019). To the best of our knowledge, a direct comparison of RNA biomarker levels in urinary and plasma EVs has not been reported before. The levels of the majority of biomarker candidates, except for NKX3-1, did not decrease in the PostOp plasma EVs, suggesting that these RNAs are released by various different organs, whereas the expression of NKX3-1 is strictly prostate-specific (Gurel et al., 2010), therefore its levels fell after prostatectomy. Together with GLO1, it showed better diagnostic performance in distinguishing PCa from BPH as compared to urinary EVs. On the contrary, in urinary EVs, the levels of 4 biomarkers—tRF-Phe-GAA-3b and tRF-Lys-CTT-5c, miR-375-3p and piR-28004 substantially decreased after the prostatectomy, showing that in urine these RNAs are contributed mostly by the prostate and/or PCa. However, none of them could distinguish PCa from BPH. This is puzzling, since at least two of them—miR-375-3p and piR-28004 were significantly overexpressed in PCa as compared to normal prostate tissues (Bajo-Santos et al., 2023). The two tRFs (tRF-Phe-GAA-3b and tRF-Lys-CTT-5c) have also been previously found as significantly overexpressed in metastatic vs*.* organ-confined disease and prognostic of biochemical recurrence (Martens-Uzunova et al., 2012; Olvedy et al., 2016). Conceivably, these RNAs are also overexpressed in BPH tissues, or the EV release rate in urine from PCa tissues is lower than that from BPH tissues. These results seem to contradict the findings from our previous study, where urinary EVs demonstrated a notably higher enrichment in PCa and/or prostate biomarker candidates (Bajo-Santos et al., 2023). This disparity most likely is attributed to the challenges encountered in normalizing urinary EV PCR results.

Out of our 15 RNA biomarker candidates, mRNAs NKX3-1 and GLO1 showed the highest diagnostic value. *NKX3-1* is an androgen-regulated gene with prostate-specific expression pattern (Gurel et al., 2010). It is located on chromosome 8p21.2, a region that is deleted in up to 86% of PCa cases (Vocke et al., 1996). It encodes a homeobox-containing transcription factor that negatively regulates epithelial cell growth and prostate morphogenesis thus functioning as a prostate-specific tumor suppressor (He et al., 1997; Papachristodoulou et al., 2021). However, we found that NKX3-1 mRNA is overexpressed in PCa tissues as compared to normal prostate tissues, and its level in plasma EVs is increased in patients with PCa as compared to BPH. We reasoned that the transcription rate of NKX3-1 is increased in androgen-sensitive PCa cells, whereas the protein functions and/or levels may be affected by mutations or post-translational modifications (Padmanabhan et al., 2016). Indeed, the analysis of transcriptomic data from TCGA database confirmed that NKX3-1 mRNA levels are increased in PCa as compared to normal prostate. In line with previous studies (Voeller et al., 1997), we did not find somatic point mutations in the coding region of *NKX3-1*, whereas we identified a pattern of SNPs suggesting a LOH affecting this gene in 4 out of 10 PCa patients analyzed. Furthermore, it is also possible that NKX3-1 mRNA is increasingly degraded and/or sorted into EVs to deplete its intracellular concentration in PCa cells. Furthermore, finding higher NKX3-1 levels in plasma EVs in patients with aggressive PCa is aligned with a previous study showing that high NKX3-1 levels in cell-free plasma are associated with aggressive PCa characteristics (De Souza et al., 2020). Additionally, higher EV-enclosed NKX3-1 levels originating from PCa cells compared to normal prostate cells had also been previously reported, aligning with our results (Lázaro-Ibáñez et al., 2017).

GLO1 codes the enzyme glyoxalase 1, a part of the glyoxalase system in the cytosol, which breaks down reactive aldehyde metabolites (Thornalley, 2003). Several studies have shown that it is overexpressed in high grade prostate cancer tissues and indicates early recurrence (Baunacke et al., 2014; Burdelski et al., 2017; Rounds et al., 2021). Despite our results not showing a statistically significant difference between GLO1 levels in high and low-grade PCa EVs, it was significantly higher in PCa patient plasma EVs than in BPH patients.

Several previous studies have explored the diagnostic and prognostic relevance of EV-enclosed RNAs in PCa patients (Ramirez-Garrastacho et al., 2022b). One of the most extensively studied biomarkers is PCA3—an lncRNA whose level in urinary EVs can differentiate between healthy men and PCa patients (Motamedinia et al., 2016), and between PCa patients with GS ≤ 6 vs*.* GS ≥ 7 (Donovan et al., 2015). Individually, PCA3 could distinguish biopsy-confirmed healthy men and PCa patients with an AUC of 0.681 (Motamedinia et al., 2016), whereas in combination with ERG mRNA and SPDEF as normalizer, it constitutes the ExoDx Prostate test that is routinely used for detection of high-grade PCa in men over 50 years of age and gray zone PSA levels (2–10 ng/mL), and informs whether to proceed with prostate biopsy (Tutrone et al., 2020). In the current study, we assessed its ability to differentiate between PCa and BPH. Although a fraction of PCa patients had elevated levels of PCA3 both in plasma and urinary EVs, its diagnostic value was moderate with AUC of 0.56 and 0.57 in plasma and urinary EVs, respectively. The best diagnostic biomarkers found in this study were GLO1 and NKX3-1 which individually could distinguish PCa from BPH in the validation cohort with an AUC of 0.726 and 0.645, respectively. Combining both biomarkers in a biomarker model yielded an AUC of 0.713 in the validation cohort, whereas adding the PSA levels to the model increased the AUC to 0.757 thus showing enhanced diagnostic performance as compared to individual markers. This observation aligns with findings from several other studies, advocating that the synergistic utilization of multiple markers is a more efficient approach to cancer detection (Prensner et al., 2012; Murphy et al., 2015; Davey et al., 2020; Ramirez-Garrastacho et al., 2022a). For instance, Davey et al. developed a seven mRNA biomarker model (FOLH1, HPN, CD24, TMPRSS2-ERG overexpressed; ITSN1, ANXA3, SLC45A3 underexpressed) that could distinguish PCa from benign conditions with AUC of 0.825. Furthermore, combining this model with two miRNAs yielded an AUC of 0.843 (Davey et al., 2020).

Taken together, in this study, we tested the diagnostic and prognostic performance of 15 RNA biomarker candidates by RT-ddPCR in independent cohorts of PCa and BPH patients. This resulted in the validation of two novel PCa biomarkers - GLO1 and NKX3-1 mRNAs that are overexpressed in PCa tissues, known to functionally contribute to the PCa development, and have higher levels in plasma EVs from PCa patients than BPH patients. In addition, higher levels of NKX3-1 in plasma and urinary EVs tended to associate with aggressive PCa. A biomarker model combining GLO1, NKX3-1 and PSA could distinguish PCa from BPH with an AUC of 0.76 in an independent validation cohort. We envision that these biomarkers could be of use for the development of liquid biopsies for the detection of clinically significant PCa, deciding on the need of prostate biopsies in men with elevated PSA levels and active surveillance of patients with low-grade disease.

## Data Availability

The original contributions presented in the study are included in the article/Supplementary Material, further inquiries can be directed to the corresponding author.

## Supplementary material

SUPPLEMENTARY IMAGE S1 Comparison of RNA biomarker levels in Pre-Op vs. Post-Op plasma EVs. Paired dot plots show copy numbers of RNA biomarkers per ml of patient plasma collected before and after radical prostatectomy in 30 PCa patients. Wilcoxon matched-pairs signed rank test was used to assess the statistical significance of the differences between groups. *p*-value < 0.05 was considered significant.

SUPPLEMENTARY IMAGE S2 Comparison of RNA biomarker levels in Pre-Op vs. Post-Op urinary EVs. Paired dot plots show copy numbers of RNA biomarkers per ml of patient urine collected before and after radical prostatectomy in 30 PCa patients. Wilcoxon matched-pairs signed rank test was used to assess the statistical significance of the differences between groups. *p*-value < 0.05 was considered significant.

SUPPLEMENTARY IMAGE S3 Comparison of RNA biomarker levels in patients with PCa vs. BPH in plasma EV samples. Violin plots show copy numbers of RNA biomarkers per ml of patient plasma in the test cohort (20 PCa, 20 BPH). Mann-Whitney test was used to assess the statistical significance of the differences between groups. *p*-value < 0.05 was considered significant.

SUPPLEMENTARY IMAGE S4 Comparison of RNA biomarker levels in patients with PCa vs. BPH in urinary EV samples. Violin plots show copy numbers of RNA biomarkers per ml of patient urine in the test cohort (20 PCa, 20 BPH). Mann-Whitney test was used to assess the statistical significance of the differences between groups. *p*-value < 0.05 was considered significant.

## Abbreviations

AUC, area under curve; BPH, benign prostatic hyperplasia; cDNA, complementary DNA; EV, extracellular vesicle; HG, high Gleason score; LG, low Gleason score; lncRNA, long non coding RNA; LOH, loss of heterozygosity; miRNA, micro RNA; mRNA, messenger RNA; NTA, nanoparticle tracking analysis; PCa, prostate cancer; piRNA, piwi-interacting RNA; PSA, prostate-specific antigen; SNP, single nucleotide polymorphism; TCGA, The Cancer Genome Atlas Program; tRF, tRNA derived fragment.
