# Supplementary material for: Validation of potential RNA biomarkers for prostate cancer diagnosis and monitoring in plasma and urinary extracellular vesicles
Source: Front Mol Biosci. 2023 Nov 30;10:1279854. doi: 10.3389/fmolb.2023.1279854 (PMC10720733; doi:10.3389/fmolb.2023.1279854)
Supplement: Supplementary file 1 [file Table1.DOCX]

***Supplementary material***

**Supplementary Table 1.** RT-ddPCR assay specifications

| Target name | Assay Type | Accession number | Annealing temperature | Description |
| --- | --- | --- | --- | --- |
| AMD1 | QuantiNova LNA | SCB0419381-200 | 55℃ | Adenosylmethionine  decarboxylase 1 |
| GLO1 | QuantiNova LNA | SCB0419367-200 | 54℃ | Glyoxalase I |
| MAZ | QuantiNova LNA | SCB0420996-200 | 54℃ | MYC Associated Zinc Finger Protein |
| NKX3-1 | QuantiNova LNA | SCB0419359-200 | 56℃ | NK3 Homeobox 1 transcript |
| PMEPA1 | QuantiNova LNA | SCB0419065-200 | 55℃ | Prostate Transmembrane Protein, Androgen Induced 1 |
| RBM47 | QuantiNova LNA | SCB0412069-200 | 56℃ | RNA Binding Motif Protein 47 |
| PCA3 | QuantiNova LNA | SCB0420991-200 | 55℃ | Prostate Cancer Associated Transcript 3 |
| PCAT14 | QuantiNova LNA | SCB0419370-200 | 54℃ | Prostate Cancer Associated Transcript 14 |
| tRNA-Lys-CTT | miRCURY LNA | YCP0047085 | 55℃ | tRNA-derived fragment Lys-CTT (315) |
| tRNA-Phe-GAA | miRCURY LNA | YCP0047088 | 55℃ | tRNA-derived fragment Phe-GAA (544) |
| miR-27a-5p | miRCURY LNA | YP00206021 | 55℃ | microRNA 27a |
| miR-92a -1-5p | miRCURY LNA | YP00204560 | 55℃ | microRNA 92a |
| miR-196a-5p | miRCURY LNA | YCP1551974 | 55℃ | microRNA 196a |
| miR-375-3p | miRCURY LNA | YCP1552280 | 55℃ | microRNA 375 |
| piR-28004 | miRCURY LNA | YCP1551995 | 54℃ | Piwi-interacting RNA 28004 |
| miR-26a-5p | miRCURY LNA | YP00206023 | 54℃ | microRNA 26a |
| let-7f-5p | miRCURY LNA | YP00204359 | 54℃ | let-7f microRNA precursor |
